# A Forgotten Rare Cause of Unilateral Basal Ganglia Calcinosis Due to Venous Angioma and Complicating Acute Stroke Management: A Case Report

**DOI:** 10.3390/diagnostics15030291

**Published:** 2025-01-26

**Authors:** Arturs Balodis, Sintija Strautmane, Oskars Zariņš, Kalvis Verzemnieks, Jānis Vētra, Sergejs Pavlovičs, Edgars Naudiņš, Kārlis Kupčs

**Affiliations:** 1 Department of Radiology, Riga Stradins University, LV-1007 Riga, Latvia; 2 Institute of Diagnostic Radiology, Pauls Stradins Clinical University Hospital, LV-1002 Riga, Latvia; kalvisverzemnieks@gmail.com (K.V.);; 3 VCA Polyclinic Plavnieki, LV-1021 Riga, Latvia; sintijasstrautmane@gmail.com; 4 Department of Neurology, Riga East Clinical University Hospital, LV-1038 Riga, Latvia; 5 Faculty of Medicine, Riga Stradins University, 16 Dzirciema Street, LV-1007 Riga, Latvia; 6 Department of Radiology, University of Latvia, LV-1586 Riga, Latvia; 7 Clinic of Neurology, Pauls Stradins Clinical University Hospital, LV-1002 Riga, Latvia; 8 Department of Neurology, Riga Stradins University, LV-1007 Riga, Latvia

**Keywords:** unilateral basal ganglia calcinosis, venous angioma, developmental venous anomalies, complicated stroke management, neuroradiology

## Abstract

**Background:** Unilateral basal ganglia calcinosis (BGC) is a rare radiological finding that can be diagnosed on computed tomography (CT) and magnetic resonance imaging (MRI) but often presents challenges for clinicians and radiologists in determining its underlying cause. So far, only a few potential causes that could explain unilateral BGC have been described in the literature. **Case Report:** A 54-year-old Caucasian male was admitted to a tertiary university hospital due to the sudden onset of speech impairment and right-sided weakness. The patient had no significant medical history prior to this event. Non-enhanced computed tomography (NECT) of the brain revealed no evidence of acute ischemia; CT angiography (CTA) showed acute left middle cerebral artery (MCA) M2 segment occlusion. CT perfusion (CTP) maps revealed an extensive penumbra-like lesion, which is potentially reversible upon achieving successful recanalization. However, a primary neoplastic tumor with calcifications in the basal ganglia was initially interpreted as the potential cause; therefore, acute stroke treatment with intravenous thrombolysis was contraindicated. A follow-up CT examination at 24 h revealed an ischemic lesion localized to the left insula, predominantly involving the left parietal lobe and the superior gyrus of the left temporal lobe. Subsequent gadolinium-enhanced brain MRI revealed small blood vessels draining into the subependymal periventricular veins on the left basal ganglia. Digital subtraction angiography was conducted, confirming the diagnosis of venous angioma. **Conclusions:** Unilateral BGC caused by venous angioma is a rare entity with unclear pathophysiological mechanisms and heterogeneous clinical presentation. It may mimic conditions such as intracerebral hemorrhage or hemorrhagic brain tumors, complicating acute stroke management, as demonstrated in this case. Surrounding tissue calcification may provide a valuable radiological clue in diagnosing venous angiomas DVAs and vascular malformations.

## 1. Introduction

Developmental venous anomaly (DVA), also known as cerebral venous angioma is a congenital venous malformation of veins draining brain parenchyma [1,2,3,4,5]. Some clinicians refer to them as caput medusae [1,2,3,4,5]. DVAs consist of a radially arranged configuration of medullary veins that are distinct from the normal brain parenchyma, forming a pattern that resembles an “umbrella” [2,3,4,5]. DVAs are a subtype of cerebrovascular malformations (CVM) that account for up to approximately 55% of such lesions, and among general population CVMs occur in 2.6 to 6.4%, relatively widely depending on the demographics, study population, region, etc. [2,3,5,6]. Mostly, these lesions are clinically insignificant, although, in the literature, they have been associated with migraine and other symptoms [3,4,5,6,7].

The majority of DVAs are diagnosed incidentally and, due to their benign clinical course, these lesions generally do not require intervention [3,5,6,7]. However, in rare, extraordinary cases, a DVA can be symptomatic, causing headaches, seizures, neurological deficits, and even cerebral infarction and hydrocephalus [3,5,6]. For instance, in 2019, Althobaiti E et al. published a case report where DVA was mimicking a thrombosed cerebral vein; in this case, the patient was admitted to a primary health care service due to a severe throbbing headache and high blood pressure [8]. Digital subtraction angiography (DSA) is considered the gold standard for the diagnosis of DVA’s; however, they can often be identified using contrast-enhanced cross-sectional imaging modalities such as computed tomography (CT), magnetic resonance imaging (MRI), or magnetic resonance angiography (MRA) [5].

Furthermore, basal ganglia calcification (BGC) is a neurodegenerative process that may occur primarily as a presentation of Fahr’s disease, secondary due to an underlying cause (Table 1) or related to aging [9]. Similarly to DVAs, BGC is characterized by its indolent nature; therefore, the diagnostic process may be challenging. Brain non-enhanced computed tomography (NECT) is considered a diagnostic gold standard when identifying calcifications as hyperdense lesions [10]. Often these calcifications are observed in the basal ganglia; however, cases with calcifications occurring in subcortical structures such as dentate nuclei, thalamus, and subcortical white matter have been previously described [10]. Less frequently, calcifications can also be found in the internal capsule, cerebral and cerebellar cortex, and brainstem [10]. Potential causes of unilateral and bilateral BGC are summarized in Table 1.

To date, only a limited number of case reports have documented unilateral BGC associated with DVAs. The majority of authors claim that DVA’s contribute to the development of unilateral BGC due to the presumed venous hypertension in the territory drained by the vascular malformation. Based on the available publications and scientific literature, pathological unilateral BGC may arise from a few potential etiologies. Comprehensive patient evaluation and tailored therapeutic interventions are crucial for achieving optimal recovery, favorable prognosis, and improved long-term outcome.

## 2. Case Presentation

A fifty-four-year-old Caucasian male was admitted to a tertiary university hospital due to a sudden speech impairment and right-sided weakness. His medical history included primary arterial hypertension (PAH), dyslipidemia, and lumbar and sacral spine spondylosis with myofascial pain syndrome. No prior ischemic events were documented. The patient’s sibling also had a history of PAH, and she had a history of an intracerebral hemorrhage at the age of 54. The neurological examination revealed mild central-type paresis of the facial muscles on the right side, mild right-sided hemiparesis, and right side hemihypesthesia. Deep tendon reflexes were more pronounced on the left side compared to the right side in both upper and lower extremities. The patient was stable in the Romberg’s maneuver. The patient exhibited pronounced motor aphasia.

Levels of stroke severity are measured according to the National Institutes of Health Stroke Scale (NIHSS) system, and these are as follows: 0 points—no stroke; 1–4—minor stroke; 5–15—moderate stroke; 16–20—moderate to severe stroke; 21 points and higher indicate severe stroke (maximum: 42 points) [19]. Neurological deficits such as paresis and speech impairment (aphasia) are considered as disabling stroke symptoms [20]. In this case, the patient scored 10 at the NIHSS including symptoms such as mild central type paresis of the facial muscles on the right side and mild right-sided hemiparesis, pronounced motor aphasia, and right-sided hemihypesthesia, indicating moderate stroke, while the mRS was 4 points, noting moderately severe disability at admission.

The patient’s clinical presentation and risk factors were indicative of acute intracerebral ischemia. In alignment with the clinical scenario, a native NECT of the brain was performed, critical in assessing eligibility for intravenous thrombolysis—a key intervention for acute ischemic stroke.

**Figure 1:**
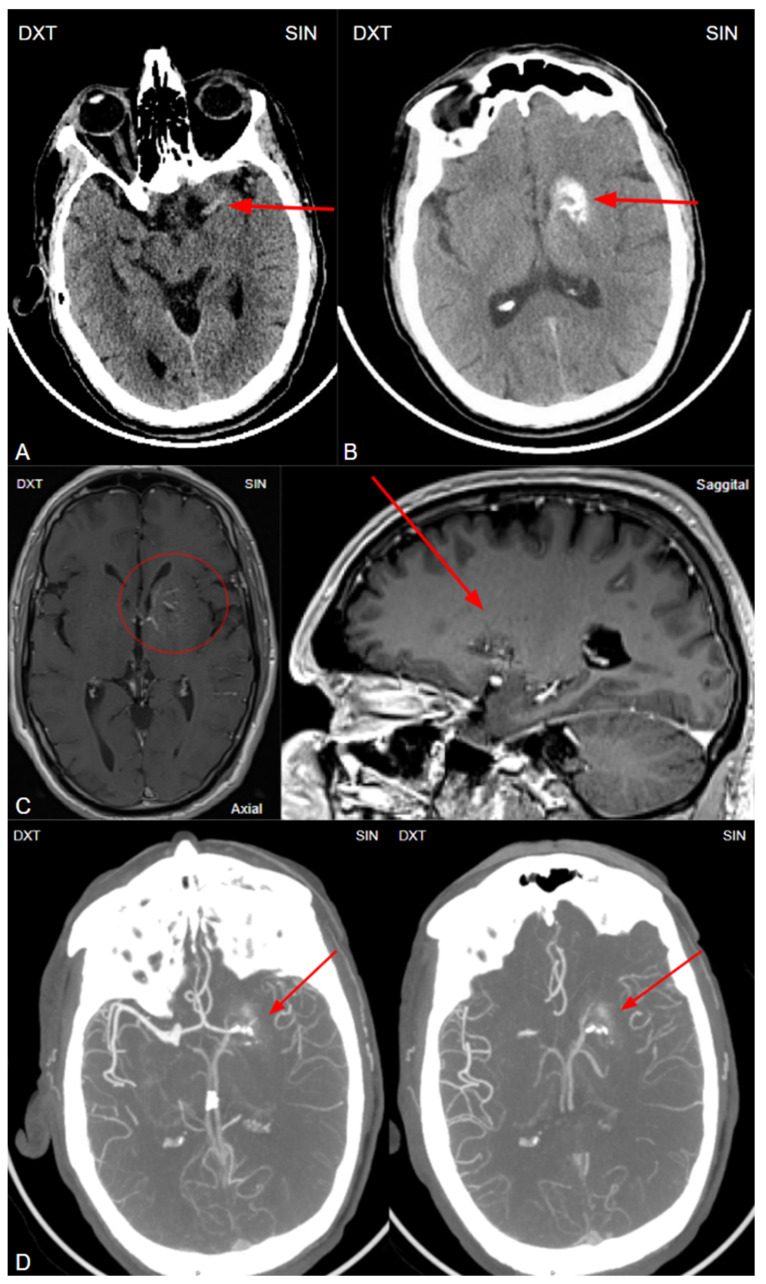
(**A**) Non-enhanced computed tomography of the brain showing a hyperdense artery sign, arteria cerebri media sin. M1 segment (red arrow). The hyperdense artery sign typically indicates acute thrombosis, especially in the presence of corresponding neurological symptoms. (**B**) Non-enhanced computed tomography of the brain at the basal ganglia level shows unilateral basal ganglia calcinosis, predominantly in the caput nuclei caudati and nucleus lentiforme (red arrow), without perifocal edema or mass effect, suggesting changes in a more likely benign nature. (**C**) Computed tomography post-contrast on left basal ganglia level in axial and sagittal planes showing a low contrast enhancement vessel venous angioma, which corresponds to developmental venous anomaly (DVA) and is regarded as the underlying cause of basal ganglia calcinosis (red circle and red arrow). (**D**) Retrospective analysis of the computed tomography angiography (MIP-CTA) images, performed before the MRI and DSA examinations, reveals a small venous angioma (DVA) in the left basal ganglia (red arrows).

**Figure 2:**
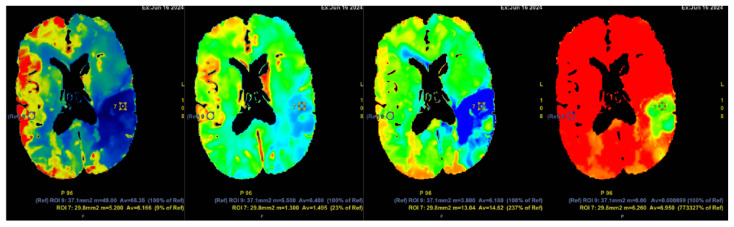
Computed tomography perfusion (CTP) after contrast injection shows a large hypoperfusion area in the territory of the left middle cerebral artery (ACM sin) with extensive penumbra-type damage (salvageable brain tissue) and a small core-type lesion in the parietal lobe, comprising less than one-third of the total hypoperfusion volume. The findings suggest the patient could potentially benefit from intravenous thrombolysis. Cerebral blood flow (CBF) 9%; cerebral blood volume (CBV) 23%; mean transit time (MTT) 237%.

The initial NECT radiological findings did not provide a definitive diagnosis and did not show any signs of acute ischemia. Considering the patient’s clinical presentation and initial NECT findings, further investigation with CT angiography (CTA) was conducted. An acute occlusion of the M2 segment of the left middle cerebral artery (MCA) was identified, accompanied by leptomeningeal collaterals extending to the left brain hemisphere with a Tan collateral score of 3 (Figure 1). A subsequent perfusion CT (CTP) was performed, revealing an extensive hypoperfusion zone predominantly consistent with a penumbra-type lesion (Figure 2). No hemorrhage was observed. The emergency department radiologist and neurologist raised concerns regarding the possibility of an acute ischemia combined with primary neoplastic tumor and BGC, potentially a high-grade oligodendroglioma. Therefore, acute thrombolytic therapy with intravenous thrombolysis was contraindicated. Thrombolysis is typically constrained to a standardized 4.5 h window to optimize reperfusion outcomes and minimize the risk of ischemia-reperfusion injury [21,22].

The patient was consulted by an interventional neuroradiologist. In the hospital where the patient was admitted, acute reperfusion therapy with intravenous thrombolysis and mechanical thrombectomy is performed daily, and it is available 24/7. A detailed assessment of vascular anatomy was conducted. Eventually, the decision to abstain from the endovascular treatment in this case was made in an interdisciplinary team including an interventional neuroradiologist, as the occlusion of the left MCA M2 segment was distal, and the extracranial vascular anatomy was rather complex.

The patient was subsequently admitted to the Stroke Unit for further evaluation and management.

**Figure 3:**
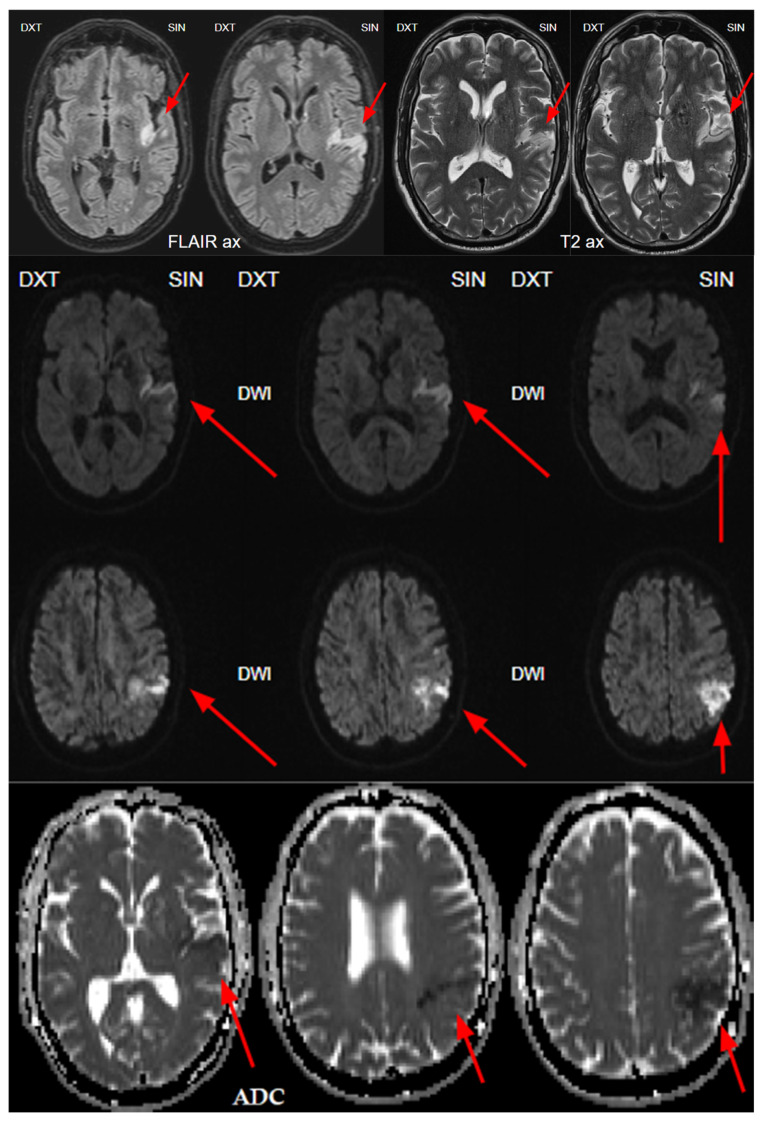
At 1 day post-admission, a magnetic resonance imaging (MRI) with fluid-attenuated inversion recovery (FLAIR) and T2-weighted sequences reveals acute ischemia in the left insula and left parietal lobe, and upper gyrus of temporal lobe corresponding to the lesion seen on CTP and consistent with the territory of the left middle cerebral artery (MCA), M2 segment (red arrows). Diffusion-weighted imaging (DWI) sequence showing restricted diffusion on left side insula, left parietal, and temporal lobe with low apparent diffusion coefficient (ADC) map value, which corresponds to acute infarction of the middle cerebral artery territory of the left side M2 occlusion (red arrows).

**Figure 4:**
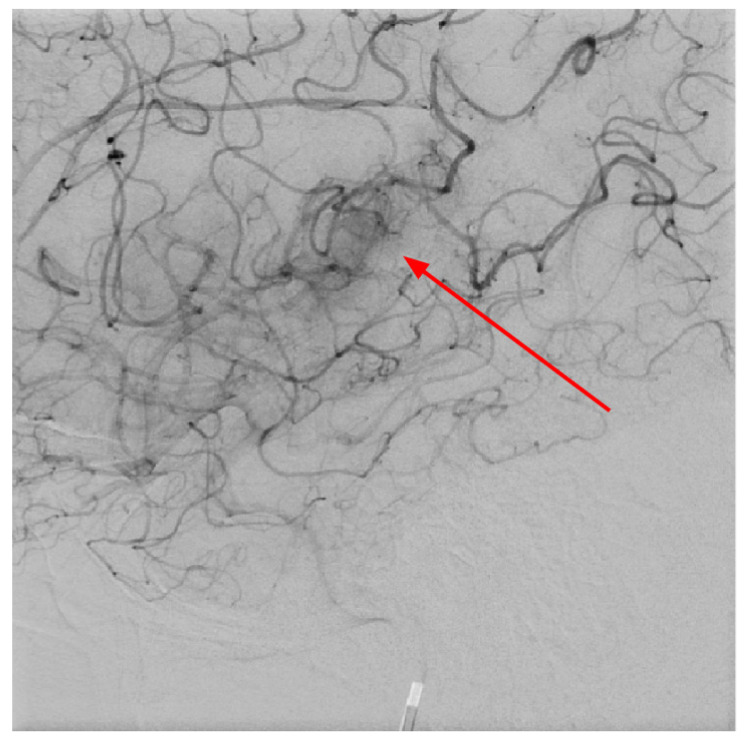
Digital subtraction angiography in LL projection at the level of basal ganglia showing abnormal vessels, which corresponds to developmental venous anomaly angioma in the left area of the basal ganglia, also these changes are seen on CTA and MRI after contrast injection (red arrow).

At 1 day post-admission, a neuroradiologist reviewed a control follow-up NECT that demonstrated an ischemic lesion localized to the left insula, predominantly involving the left parietal lobe and the superior gyrus of the left temporal lobe (Figure 1). Additional radiological findings included a hyperdense artery sign, characteristic of acute thrombosis, and BGC on the left side, warranting further investigation to clarify the underlying etiology. Subsequently, MRI of the brain was conducted, which also revealed ischemic signs, as well as unilateral BGC (Figure 3). DSA was performed to clarify reasons for unilateral BGC, and it confirmed the presence of a DVA (Figure 4).

Conservative treatment was initiated, including antiplatelet therapy with aspirin, an angiotensin receptor blocker (valsartan), a thiazide diuretic (hydrochlorothiazide), and HMG-CoA reductase inhibitors (statin) rosuvastatin. To address post-infarction neurological complications, such as motor aphasia, right-sided hemihypesthesia, mild hemiparesis, facial muscle paralysis, and fine motor impairment, a multidisciplinary team with functional specialists including a speech therapist, physiotherapist, and ergotherapist was engaged. The patient was closely monitored over the next few days. Neurological symptoms gradually improved, and the patient was discharged from the hospital in a good overall health condition five days after symptom onset, with further recommendations to continue the prescribed pharmacological therapy and physiotherapy at home.

## 3. Discussion

DVAs and BGC have been previously described in the literature. DVAs are composed of dilated medullary veins draining normal brain parenchyma, ultimately draining into the deep or the superficial venous system [23]. DVAs are thought to arise from a maldevelopment of fetal cortical venous drainage, but the clear etiology of DVAs is still under debate [24]. Usually, DVAs are benign and incidentally discovered by either angiographic or contrast-enhanced brain MRI, usually resembling “caput medusae” [1,2,7,24]. However, the number of publicly-available case reports about unilateral BGC due to venous angioma is under 20.

In 2010, Dehkharghani et al. published an article demonstrating six case reports with unilateral caudate and putamen calcifications in DVA drainage territories. In all these patients, DVA was found in gadolinium-enhanced MRI and/or CTA or conventional angiography. They stressed the venous hypertension as the main contributing factor for these abnormalities [25]. Moreover, they reported no symptoms referable to the basal ganglia, and the patients they presented did not reveal underlying metabolic disorders or processes associated with calcium deposition [25]. In our case, the patient was a fifty-four-year-old male presenting with a sudden speech impairment and right-sided weakness. No abnormal movements were noted in this patient. A subacute stroke on the left side in the dorsal part of the insula, in the upper dorsal part of the left temporal lobe, and partially in the left parietal lobe was found on NECT, and CTA revealed left MCA M2 occlusion. In CTP observation, markedly decreased CBF and CBV was noted along with increased MTT. Subsequently, gadolinium-enhanced brain MRI was performed, where small blood vessels draining to subependymal periventricular veins on T1 post-contrast was found. Due to these findings, the patient underwent the following DSA, where venous angioma in the area of the left basal ganglia was observed.

Another possible cause of unilateral BGC could be oligodendrogliomas (OG). OGs are rare neuroepithelial tumors of the central nervous system, a type of glioma, accounting for up to 5% of primary intracranial neoplasms [18]. These tumors are predominantly found in the frontal lobes of the brain, often involving the cortical or subcortical regions [16,18]. Most OGs contain coarse calcification, observed in 30% to 90% of cases [16,17,18]. The presence of calcification is characterized by the high signal intensity in T2-weighted MRI sequences [16,17,18]. In radiological imaging, these calcifications could potentially present unilaterally, depending on the OG’s localization [17,18,19].

Unilateral BGC may also be associated with non-ketotic hyperglycemia (NKH), hyperglycemic hemichorea syndrome, also known as diabetic striatopathy, or chorea-hyperglycemia-basal ganglia (C-H-BG) syndrome. This syndrome is often described to have a typical triad that involves unilateral involuntary movements, contralateral striatal abnormality on neuroimaging, and the resolution of symptoms after correction of hyperglycemia [14,15]. Brain NECT and MRI may demonstrate unilateral hyperintensity in the striatal region, most commonly the putamen, which can manifest contralaterally in the body as hemiballism or hemichoreic movements [14,15].

Sometimes, AVMs may occur in patients with genetic conditions [26]. Just recently, in October 2024, Toader et al. published a case report of a 36-year-old female with prothrombin G20210A mutation-associated thrombophilia, highlighting its potential impact on AVM pathophysiology and management [26]. In this case, the patient underwent frontal craniotomy with microsurgical resection of the AVM, which proved to be a viable and effective treatment option [26].

In 2022, Patel et al. presented a clinical case report about a patient with hyperkinetic choreiform movements attributed to BGC and underlying DVA [27]. Movement disorders are often associated in the setting of a recent stroke, but chorea-like hyperkinetic movements in association with DVAs have not been thoroughly reported so far [27,28]. Their imaging results revealed right-sided unilateral BGC associated with DVA in the absence of stroke, contributing to contralateral hyperkinetic movements [27]. Another publication about unilateral hyperkinetic choreiform movements due to BGC and underlying DVA was published in 2019 by Falconer et al. [29]. They pointed out clonazepam as one of the treatment options for such cases [29]. Patel et al. revealed a trial with clonazepam in their patient, resulting in almost near-resolution of hyperkinetic movements [27].

Intracerebral calcifications may be misinterpreted as acute intracerebral hemorrhage (ICH) [30,31]. Brain MRI could offer a diagnostic advantage over CT imaging in differentiating calcifications from a small ICH, which may not be accompanied by localized edema. A prospective, single-center study in patients with spontaneous ICH reported that MRI was slightly more sensitive than CT for detecting small intraventricular hemorrhage, where MRI sensitivity was 100% compared with 97% for CT [32]. ICH is a life-threatening type of stroke, characterized by bleeding into the brain parenchyma. This type of stroke accounts for approximately 15% of all strokes, and it occurs in younger patients compared with ischemic stroke patients [33,34]. Depending on the size and location of the ICH, the clinical picture may be extremely variable. Sudden, severe headache, also known as thunderclap headache, is the most common presentation, but other symptoms may include nausea, vomiting, seizures, confusion, altered consciousness and mental status, motor deficits, aphasia, personality changes, dizziness, slurred speech, vertigo, etc. [35].

It is crucial to accurately differentiate intracerebral calcification and its etiology from acute pathologies such as ICH and intracerebral hemorrhagic neoplasms, as well as previously mentioned NKH and DVA, to perform adequate therapeutic interventions.

In our patient, no abnormal movements were noted. During his hospitalization, the patient underwent a wide range of tests and exams, revealing no data of systemic or genetic disorders. The patient received conservative treatment. However, mild motor aphasia, slight coordination impairment in the right upper extremity and sensory ataxia in both lower extremities persisted along with deep tendon reflex asymmetry in both arms and in both legs: dx > sin. After 5 days, the patient was discharged from the hospital in a good overall health condition, with further recommendations to continue antiplatelet therapy with aspirin indefinitely, antihypertensive therapy, and rosuvastatin.

This clinical case report demonstrates a highly valuable and educative radiologic finding about unilateral BGC and DVA, considering that there are less than 20 case reports available so far. This report is anticipated to be of significant interest and value to medical professionals, particularly for radiologists and multidisciplinary stroke teams. Unilateral BGC is a rare pathology with only a few differential diagnoses. This article highlights the importance of accurately identifying this pathology, as calcification may masquerade as other pathologies, potentially leading to unnecessary alterations in therapeutic strategies. Such alterations may detrimentally impact the patient’s quality of life and lead to severe complications, particularly in individuals with concurrent comorbidities, such as stroke in this instance. This clinical case report provides an additional contribution to the science of this pathology, however there is still a need for more research and awareness of this pathology to increase the knowledge about such cases and improve patient management.

## 4. Conclusions

To conclude, unilateral BGC due to venous angioma is a rare condition characterized by unclear pathophysiological mechanisms, a limited number of potential etiologies, and a heterogeneous clinical presentation. It can mimic other conditions, such as intracerebral hemorrhage or hemorrhagic brain tumors, thereby complicating acute stroke management, as illustrated in this case. Comprehensive interdisciplinary patient evaluation and therapeutic interventions are crucial to achieving optimal recovery and improved long-term outcome.

## Figures and Tables

**Table 1:** Causes of bilateral basal ganglia calcification [3,11,12,13].

Idiopathic	Age, Fahr’s disease-primary familial brain calcification
Toxic	Carbon monoxide intoxication, lead intoxication, mineralizing microangiopathy (radiation/chemotherapy), nephrotic syndrome, vitamin D intoxication, excess calcium intake, methotrexate therapy
Infectious	TORCH infections (Toxoplasmosis, Syphilis, Varicella Zoster Virus, Parvovirus B19, Rubella Virus, Citomegalovirus, Human Simplex Virus), Epstein–Barr Virus, tuberculosis, human immune virus, parasitic invasion (Cysticercosis, Cystic Echinococcosis)
Inflammatory Diseases	Systemic lupus erythematosus
Endocrine Disorders	Hypoparathyroidism, pseudohypoparathyroidism, pseudopseudohypoparathyroidism, hyperparathyroidism, hypothyroidism, Addison’s disease
Neoplastic Disorders	Ependymoma, oligodendroglioma, mucinous adenocarcinoma
Metabolic Disorders	Mitochondrial diseases, phenylketonuria Type 2, sulfocysteinuria, GM1 Gangliosidosis, dihydropteridine reductase deficiency
Congenital Disorders	Cockayne syndrome, Down syndrome, tuberous sclerosis, lipoid proteinosis (hyalinosis cutis), methemoglobinemia, Sanjad– Sakati syndrome, Aicardi–Goutières syndrome, oculodentodigital dysplasia, congenital dyskeratosis, cerebrooculo-facio-skeletal syndrome
Neurodegenerative Diseases	Hallervorden–Spatz syndrome, neuroferritinopathy, dentatorubral–pallidoluysian atrophy
Ischemia	Hypoxia, asphyxia, neonatal hypoxia, cardiovascular events
Vascular pathologies	Developmental venous anomalies, hematomas
Potential causes of unilateral basal ganglia calcification [3,14,15,16,17,18].	
Metabolic Disorders	Non-ketotic hyperglycemia
Neoplastic Disorders	Oligodendroglioma
Vascular pathologies	Developmental venous anomalies

## Data Availability

The original contributions presented in the study are included in the article, further inquiries can be directed to the corresponding author.
